# A Transformers-based framework for refinement of genetic variants

**DOI:** 10.3389/fbinf.2025.1694924

**Published:** 2026-01-05

**Authors:** Omar Abdelwahab, Davoud Torkamaneh

**Affiliations:** 1 Département de Phytologie, Université Laval, Québec, QC, Canada; 2 Institut de Biologie Intégrative et des Systèmes (IBIS), Université Laval, Québec, QC, Canada; 3 Centre de recherche et d’innovation sur les végétaux (CRIV), Université Laval, Québec, QC, Canada; 4 Institut intelligence et données (IID), Université Laval, Québec, QC, Canada

**Keywords:** bioinformatics & computational biology, deep learning, artificial intelligence, genomics, transformers, variant calling analyisis, variant filtering, VCF analysis

## Abstract

Accurate variant calling refinement is crucial for distinguishing true genetic variants from technical artifacts in high-throughput sequencing data. While heuristic filtering and manual review are common approaches for refining variants, manual review is time-consuming, and heuristic filtering often lacks optimal solutions, especially for low-coverage data. Traditional variant calling methods often struggle with accuracy, especially in regions of low read coverage, leading to false-positive or false-negative calls. Advances in artificial intelligence, particularly deep learning, offer promising solutions for automating this refinement process. Here, we present a Transformers-based framework for genetic variant refinement that leverages self-attention to model dependencies among variant features and directly processes VCF files, enabling seamless integration with standard pipelines such as BCFTools and GATK4. Trained on 2 million variants from the GIAB (v4.2.1) sample HG003, the framework achieved 89.26% accuracy and a ROC AUC of 0.88. Across the tested samples, VariantTransformer improved baseline filtering accuracy by 4%–10%, demonstrating consistent gains over the default caller filters. When integrated into conventional variant calling pipelines, VariantTransformer outperformed traditional heuristic filters and, through refinement of existing caller outputs, approached the accuracy achieved by state-of-the-art AI-based variant callers such as DeepVariant, despite not operating as a standalone caller. By positioning this work as a flexible and generalizable framework rather than a single-use model, we highlight the underexplored potential of Transformers for variant refinement in genomics. This study contributes a blueprint for adapting Transformer architectures to a wide range of genomic quality control and filtering tasks. Code is available at: https://github.com/Omar-Abd-Elwahab/VariantTransformer.

## Introduction

Genetic variants are considered the backbone for identifying genomic diversity, detecting disease-associated mutations, and enabling population-level genetic studies, and are fundamental to genetic screening tools (Syvänen, 2001). Variant calling is the process of identifying differences between an individual’s genome and a reference genome, encompassing single nucleotide polymorphisms (SNPs), small insertions and deletions (InDels), and structural variations (Green et al., 2011). This study focuses specifically on the refinement of small variants (SNPs and InDels). However, the raw output of variant calling pipelines often contains technical artifacts, false positives (FPs), and low-confidence calls, particularly in regions of low coverage or high complexity. As a result, variant refinement—a critical post-calling stage—is required to distinguish true variants from noise and to ensure that downstream analyses are based on reliable genetic information (Van der Auwera et al., 2013; Hemstrom et al., 2024).

Typically, variant refinement involves heuristic filtering and/or manual review. Heuristic filtering entails establishing project-specific thresholds for key metrics such as read depth, variant allele fraction (VAF), base quality, read quality, and mapping quality scores (Carson et al., 2014). While fast, these thresholds are context-dependent, often suboptimal, and can vary widely across projects (Lefouili and Nam, 2022; Pfeifer, 2016; Li and Durbin, 2009; Langmead and Salzberg, 2012). Overly strict thresholds increase false negatives (FNs), while lenient thresholds inflate FPs. Manual review, while time-intensive and not scalable for large variant sets, enhances confidence in specific variants by uncovering patterns typically overlooked by conventional variant callers through direct visual inspections of the variants using genomic viewers like Integrative Genomic Viewer (IGV) (Robinson et al., 2011; Robinso et al., 2017). Despite its importance, the refinement process in variant calling is often underdeveloped and lacks comprehensive representation in genomic workflows.

With the emergence of artificial intelligence (AI), new models have been introduced to enhance the process of variant calling refinement (Spinella et al., 2016; Ding et al., 2012; Ainscough et al., 2018). These early efforts have shown potential in utilizing machine learning (ML) and deep learning (DL) techniques to improve the precision of variant analysis and refinement. However, many of these tools are tightly coupled to specific pipelines, sequencing depths, or variant types, which can limit their generalizability in some contexts. For example, while DeepVariant has demonstrated strong cross-platform and cross-coverage performance, it remains computationally intensive and primarily optimized for high-coverage datasets. In particular, low-coverage sequencing data (≤15×)—common in population genomics, crop genomics, or large-scale human studies—remains a challenging setting where heuristic filters struggle and where many existing AI models are not optimized (Strom, 2016).

Transformers, a revolutionary class of DL models originally developed for natural language processing (NLP), have demonstrated exceptional capabilities in identifying complex patterns and dependencies in sequential data (Vaswani et al., 2017). Their unique architecture, which includes “self-attention” and “feed-forward neural network” layers, allows for dynamic learning of correlations among features, thereby enhancing classification tasks (Vaswani et al., 2017). Their self-attention mechanism allows the model to weigh relationships between features dynamically, making them highly adaptable to structured yet noisy input data—such as variant feature tables extracted from Variant Calling Format (VCF) files. Given their adaptability, Transformers are ideally suited to address the challenges of variant calling refinement, enabling the thorough analysis of extensive genomic datasets and facilitating the extraction of high-quality genomic variations.

In this study, we introduce VariantTransformer, a general Transformers-based framework for the refinement of genetic variants. While we demonstrate its effectiveness on low-coverage Illumina datasets from the Genome in a Bottle (GIAB) project, the framework is designed to be dataset-agnostic and model-agnostic: it can be trained or fine-tuned on VCF outputs from any variant caller (such as BCFTools (Danecek et al., 2021) and GATK (McKenna et al., 2010)), sequencing platform, or coverage level. However, because different variant callers encode partially distinct INFO field structures, cross-caller adaptation may require adding new tokens—representing additional INFO attributes or encoded words from the new feature set—before retraining or fine-tuning the model. Each variant record is treated as a tokenized “sentence,” enabling us to cast refinement as a binary classification task (PASS/FAIL). The predicted labels are then directly written to the FILTER column of the VCF, making the approach immediately compatible with standard bioinformatics pipelines. VariantTransformer updates only the final PASS/FAIL decision in the FILTER field. Any pre-existing FILTER annotations produced by the variant caller (e.g., depth filters, strand bias flags, platform-specific tags) are preserved and appended to the model’s output. This ensures compatibility with downstream tools that rely on caller-defined FILTER metadata.

## Methods

### Sequencing data

The FASTQ files containing sequencing data, generated on an Illumina HiSeq2500, for three samples (HG003, HG006, and HG007) with sequence coverages of 10.5X, 13.6X, and 12.6X, respectively, were procured from the GIAB Consortium (Zook et al., 2016), accessed via the NIST GIAB FTP site (https://ftp-trace.ncbi.nlm.nih.gov/giab/ftp/data/). We used SAMtools (Li et al., 2009) to determine the coverage of samples, utilizing the ‘-a’ option, to consider all positions within the reads. The alignment of the raw FASTQ files to the human reference genome GRCh38 (GCA_000001405.15_GRCh38_no_alt_analysis_set.fna) was performed using Sentieon BWA-MEM (Freed et al., 2017). The exact VCFs used for training and evaluation are publicly available in Zenodo (DOI: https://doi.org/10.5281/zenodo.17794617).

### Training and testing data

Variant calling on the aligned BAM files was performed with GATK4 HaplotypeCaller (McKenna et al., 2010) and BCFTools (Danecek et al., 2021). To ensure accurate variant classification, we used the latest GIAB truth sets v4.2.1 (Wagner et al., 2022) to update the “FILTER” column in the VCFs. We developed a custom Python script for comparing VCF files against truth sets. The script performs position-based matching: if a variant’s genomic position (POS) is present in both the test VCF and the reference VCF from the truth set, its FILTER field is set to ‘PASS’; otherwise, it is labeled as ‘FAIL’. This approach provides a consistent and transparent framework for labeling variants for model training and evaluation.

For completeness, we note that tools such as hap.py (Krusche et al., 2019) and rtg vcfeval (Cleary et al., 2015) provide alternative comparison strategies that account for representation differences between variant records. These tools can be incorporated into the framework when representation-aware benchmarking is needed.

We conducted preliminary trials to assess the effect of incorporating locus-specific information (“CHROM,” “POS,” “REF,” and “ALT”) on the model’s refinement accuracy. These features were initially considered to provide genomic context that might capture region- or allele-specific biases. However, their inclusion yielded negligible improvement in overall accuracy while substantially increasing input dimensionality and training time.

We hypothesize that this outcome stems primarily from feature engineering rather than architectural constraints. Because CHROM and POS are numerical coordinates rather than contextual features, their direct inclusion provides minimal information about local genomic context unless coupled with external annotations (e.g., repetitive regions or GC content). Similarly, REF and ALT alone are insufficient to capture sequence-level dependencies in the absence of flanking sequence data. Therefore, the exclusion of these features reflects a design choice aimed at maintaining a generalizable, lightweight framework focused on quality- and evidence-based attributes that are consistently available across callers.

This behavior is consistent with observations from prior studies showing that sequence-context or coordinate-based features tend to contribute minimally to model performance when other quality-based features are already present (Spinella et al., 2016; Ding et al., 2012; Ainscough et al., 2018). Given these findings, and to preserve computational efficiency and general applicability across diverse datasets, we excluded these fields from the final framework configuration. Nonetheless, future work could explore their potential role in more complex or repetitive genomic regions.

During preprocessing, we simplified the dataset by removing non-essential columns (“Chrom,” “POS,” “REF,” and “ALT”) and consolidating the remaining data into a single column. This restructuring facilitated the transformation of the dataset into a sentence classification format, where the merged column represented the ‘sentences’ and the “FILTER” column represented the target labels.

For training purposes, we merged the updated VCFs (generated from GATK4 and BCFTools) from the sample HG003. To reduce computational cost in the training process, we randomly selected 2 million variants from the merged VCF for initial model training and validation. Table 1 demonstrates the number of variants generated at each step. The selected dataset was then split into 60% for training and 40% for validation, with the remaining variants of the HG003 and the other VCFs from the other samples being used for further testing. HG003 was selected as the training/validation sample as a representative high-confidence GIAB genome. This choice provides a straightforward and conventional setup for demonstrating the VariantTransformer framework. However, the approach is not tied to this specific sample: users may retrain or fine-tune the model on any genome, population, or truth set according to their study design.

**TABLE 1:** Breakdown of “PASS” and “FAIL” variants from sample HG003, processed with GATK4 and BCFTools.

HG003 sample	PASS	FAIL	Total
BCFTools	3,714,910	708,823	4,423,733
GATK4	3,668,708	964,363	4,633,071
Total_merged_file	7,383,618	1,673,186	9,056,804
Training and validation set	1,630,866	369,134	2,000,000

In the testing process, we used two other samples (HG006 and HG007) (Table 2). We tested in batches of 10,000 to generate probabilities for further model performance analyses.

**TABLE 2:** Variant counts obtained from samples HG006 and HG007 via GATK4 and BCFTools, detailing “PASS” and “FAIL” variants.

Sample	Coverage (X)	Variant caller	PASS	FAIL	Total
HG006	13.6	BCFTools	3,694,662	826,173	4,520,835
		GATK4	3,666,347	1,066,280	4,732,627
HG007	12.6	BCFTools	3,685,056	847,135	4,532,191
		GATK4	3,650,142	1,042,385	4,692,527

While we excluded locus-specific columns (CHROM, POS, REF, ALT) to reduce complexity for this initial implementation, the framework is modular. Additional features such as read-level statistics, platform-specific quality scores, or caller-specific annotations can be incorporated as tokens without altering the core Transformer structure. This design makes the framework adaptable to diverse datasets and sequencing technologies.

### Framework development and analysis

The framework is instantiated here with a BERT-based Transformer architecture (Vaswani et al., 2017); however, it is not tied to a specific implementation. Alternative Transformer backbones (e.g., RoBERTa, ALBERT, or lightweight genomic Transformers) can be substituted with minimal adjustments. This flexibility underscores that our method is a generalizable blueprint for variant refinement rather than a fixed architecture.

A DL framework was developed based on the Transformers architecture (Vaswani et al., 2017) to automate the variant calling refinement process. We used the BertForSequenceClassification model from Hugging Face (Wolf et al., 2019) while tuning some of the parameters in the configuration and tokenization steps to achieve better performance. For parameter tuning, we initially conducted a grid search to identify optimal hyperparameters. Key parameters adjusted included the learning rate (0.00005–0.0001), batch size (600–1,300), number of attention heads (6–12), and number of hidden layers (6–12). We also integrated domain-specific vocabulary into the BertTokenizer, which allowed the model to better interpret genomic context. The final configuration was chosen based on the highest ROC AUC score during validation. Further details regarding the configuration parameters are available in Supplementary Table S1. For the rest of the parameters, we used the default values mentioned in the Hugging Face documentation (Wolf et al., 2019). The model was trained over 21 epochs with a batch size of 1,300 using the AdamW optimizer (Loshchilov and Hutter, 2017), focusing on balancing performance and resource utilization.

The framework can be scaled for integration into large-scale genomic pipelines. In practice, VariantTransformer can be further optimized by adopting lighter Transformer variants or adjusting batch strategies, depending on the computational environment.

### Evaluation metrics

To thoroughly evaluate framework performance, we employed several accuracy metrics, including the AUC (Area Under the Curve), ROC (Receiver Operating Characteristics) curve (Hand and Till, 2001), Matthews correlation coefficient (MCC) (Gorodkin, 2004; Baldi et al., 2000; Chicco and Jurman, 2020; Jurman et al., 2012), accuracy, precision, recall, and F1 score (Godbole and Sarawagi, 2004). Accuracy is calculated as the proportion of variant records for which the model’s predicted PASS/FAIL label matches the reference PASS/FAIL label used during evaluation. ROC and AUC were computed using the model’s predicted PASS/FAIL probability as the continuous decision score. For further framework evaluation, we reported MCC, also known as the phi coefficient, where a coefficient of +1 indicates an ideal prediction, 0 signifies an average random prediction, and −1 denotes a reverse prediction (Baldi et al., 2000; Jurman et al., 2012). All accuracy metrics were generated using scikit-learn (Varoquaux et al., 2015). Moreover, we compared the framework performance to default filtering parameters of conventional variant callers (BCFTools and GATK4), an AI-based variant caller (DeepVariant (Poplin et al., 2018)), and an AI-based tool for refinement of somatic variant calling (DeepSVR (Ainscough et al., 2018)), considering both PASS and FAIL variants. For BCFTools and GATK4, we integrated VariantTransformer into each pipeline and compared the performance of the framework against default filters. For GATK4, the default filters were QD < 2.0, FS > 60.0, MQ < 40.0, SOR > 4.0, MQRankSum < −12.5, and ReadPosRankSum < −8.0. For BCFTools, we applied only the QUAL > =20 filter. As for DeepVariant, we compared the performance of each model-integrated conventional variant caller against DeepVariant. DeepSVR was compared against VariantTransformer in terms of data preparation, multiple accuracy metrics, computational complexity, and user experience. All plots were generated using the MatPlotlib library (Hunter, 2007) or ggplot2 (Wickham, 2016).

### Clarification on GIAB resources and labeling strategy

In this study, we used the GIAB benchmark sets as a source of high-quality labels for developing a machine-learning–based filtering framework. While GIAB defines high-confidence BED regions in which variants can be reliably interpreted as true positives (TPs) or FPs, the objective of our study was not to perform GIAB-certified benchmarking. Instead, GIAB served as a consistent and well-curated reference from which VariantTransformer could learn generalizable patterns of variant quality.

For methodological coherence, we adopted a single unified labeling and comparison strategy based on positional agreement and the FILTER field from the caller VCFs. This approach ensures that the assumptions used during training are identically applied during evaluation, avoiding discrepancies that arise when training and testing use different correctness definitions.

We emphasize that the reported metrics therefore represent model filtering performance, not formal GIAB FP/FN rates. The framework is intentionally flexible: users may substitute alternative truth sets, high-confidence region definitions, or GA4GH-compliant benchmarking tools (e.g., hap. py or rtg vcfeval) without modifying the underlying methodology.

## Results

### Framework development results

**FIGURE 1:**
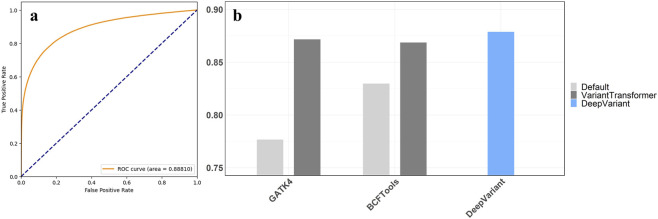
**(a)** ROC AUC curve for the trained model. **(b)** VariantTransformer’s average accuracy performance, calculated as the mean accuracy across the two test samples (HG006 and HG007) and both variant calling pipelines (GATK4 and BCFTools). The results are benchmarked against default heuristic filters and the AI-based caller DeepVariant.

VariantTransformer was developed using a dataset of 2 million variants, including both SNPs and InDels, sourced from the GIAB sample HG003. The variants were called using GATK4 and BCFTools. Of the two million variants, 1,630,866 matched the latest GIAB truth sets v4.2.1 and were classified as “PASS”, while the remaining 369,134 were classified as “FAIL”. To avoid overfitting, the data was randomly split into 60% for training and 40% for validation. The framework achieved an accuracy of 89.26% and an ROC AUC score of 0.88 (Figure 1a), demonstrating that Transformers can effectively learn feature dependencies within variant call data and are well-suited for post-calling refinement tasks.

To better highlight the advantages of VariantTransformer, we compared its performance to traditional heuristic filtering approaches using default parameters from GATK and BCFTools. As shown in Table 3, VariantTransformer outperformed traditional methods in terms of precision, recall, and F1-score, particularly in low-coverage regions (10–15X), where traditional heuristics tend to yield higher false-positive rates. Figure 1b provides a visual representation of this comparative analysis, illustrating the framework’s accuracy in refining variants compared to DeepVariant. The average accuracy shown in Figure 1b represents the mean value across both GIAB test samples (HG006 and HG007) for outputs from GATK4 and BCFTools.

**TABLE 3:** Performance of Variant Transformer across different metrics in the variant calling pipelines for samples HG006 and HG007.

Sample	Coverage (X)	Variant caller	Accuracy^a^	Refined accuracy^b^	Precision	Recall	F1score	ROC AUC	MCC
HG006	13.6	BCFTools	83.31%	87.03%	0.85957	0.87027	0.85845	0.86413	0.51194
		GATK4	77.50%	87.38%	0.87004	0.87375	0.86385	0.88876	0.60805
HG007	12.6	BCFTools	82.63%	86.68%	0.85635	0.86678	0.85197	0.83063	0.50117
		GATK4	77.82%	86.94%	0.86646	0.86944	0.85692	0.85605	0.58638

^a^ Default filtering.

^b^ After Variant Transformer refinement.

While demonstrated here on BCFTools and GATK outputs, the framework is caller-agnostic. It can be retrained or fine-tuned on VCFs generated by other callers such as DeepVariant, Strelka2, or Clair3.

The performance results presented here reflect VariantTransformer’s predictive filtering behavior under the unified labeling strategy used throughout model development. Because this evaluation does not restrict comparisons to the GIAB high-confidence regions, these metrics should not be interpreted as GIAB-standard variant-calling accuracy. Instead, they illustrate how consistently the model predicts variant quality under the same assumptions used during training, which aligns with the primary objective of demonstrating a generalizable filtering framework.

### Framework evaluation across variant calling pipelines

To test VariantTransformer applicability, we integrated it into two conventional variant calling pipelines (BCFTools and GATK4). We processed the two GIAB samples HG006 and HG007 through these pipelines and subsequently applied VariantTransformer to refine the variant calls. The framework significantly outperformed the default threshold-based filtering, achieving an overall accuracy of 87%, compared to 78% for GATK4 and 83% for BCFTools, thus approaching the performance of the AI-based variant caller, DeepVariant, which has an accuracy of 88% (Figure 1b).

During the testing phase, batches of 10,000 variants were processed to generate performance metrics such as MCC scores, ROC AUC scores, accuracy, precision, recall, and F1score. The aggregated results, outlined in Table 3, provide a comprehensive evaluation of VariantTransformer’s efficacy when integrated into the aforementioned variant calling pipelines. VariantTransformer improved both accuracy and MCC, demonstrating a more balanced classification of true and false variants. The positive MCC values (ranging from 0.50 to 0.61) confirm that the model performs substantially better than random or heuristic-based filters, particularly in low-coverage datasets. This is especially relevant for imbalanced datasets, where MCC provides a more robust measure of classifier reliability than accuracy or F1 score (Chicco and Jurman, 2020).

### Comparative analysis: assessing performance against existing pipelines and models

Our assessment included a detailed analysis of variants categorized as “FAIL.” This was done to understand the types of errors that VariantTransformer aims to address, such as borderline cases with ambiguous quality metrics. This comprehensive evaluation provides deeper insights into the framework’s robustness in filtering both “PASS” and “FAIL” variants, thus ensuring a balanced representation of TPs and true negatives (TNs).

While many benchmarking workflows report metrics primarily on PASS variants, in our study we evaluated all variants, including those labeled as FAIL, to remain consistent with the label structure used during model training. This comprehensive approach considers all four parameters: TPs, FPs, TNs, and FNs.

In our evaluation, variants labeled as “FAIL” refer to those not present in the GIAB high-confidence truth set. This binary classification follows the GIAB convention, where all variants in the reference set are considered TPs (“PASS”), and any variant absent from it is labeled as “FAIL.” This definition provides a consistent, reproducible basis for evaluating the framework’s performance. Users can readily adapt this labeling criterion for other truth sets or custom datasets when retraining or fine-tuning VariantTransformer.

The performance of VariantTransformer was compared with BCFTools and GATK4 pipelines, and with DeepVariant, focusing on two GIAB samples (HG006 and HG007) with coverage of 13.6X and 12.6X (Table 4). For sample HG006 using BCFTools, from 4,520,835 variants, VariantTransformer identified 3,993,987 as “PASS” with an accuracy of 87.03%. When using GATK4 on the same sample, the model called 4,027,338 as “PASS” with an accuracy of 87.38%. For sample HG007 using BCFTools, VariantTransformer identified 4,044,040 as “PASS” with an accuracy of 86.68%. When using GATK4 on HG007, the model called 4,060,472 as “PASS” with an accuracy of 86.94%. Comparatively, DeepVariant demonstrated slightly higher overall accuracy, with 87.95% for HG006 and 87.78% for HG007. While DeepVariant exhibited a higher number of variants labeled as FPs against the GIAB truth sets, this likely reflects its greater sensitivity—particularly in complex or repetitive genomic regions—rather than systematic overcalling. The GIAB truth sets, while highly curated, may not fully capture all true variants in such regions, meaning that some variants uniquely identified by DeepVariant could represent genuine positives outside benchmark regions. In contrast, VariantTransformer maintains a more conservative balance between sensitivity and precision, emphasizing reliable variant refinement rather than maximal detection. Notably, the values reported here reflect performance under our positional evaluation strategy; representation-aware benchmarking tools would yield metrics based on different matching criteria. Because this evaluation is based on low-coverage WGS, the absolute performance values are naturally lower than those reported in high-coverage benchmarking studies, reflecting the inherent difficulty of variant interpretation under reduced read depth.

**TABLE 4:** Detailed comparison of Variant Transformer’s performance when integrated with GATK4 and BCFTools against DeepVariant.

Sample	Coverage (X)	Variant caller	Input^a^	PASS^b^	TP	FP	FN	TN	Accuracy
HG006	13.6	BCFTools	4,520,835	3,993,987	3,551,084	442,903	143,576	383,270	87.03%
		GATK4	4,732,627	4,027,338	3,548,098	479,240	118,249	587,039	87.38%
		DeepVariant	6,643,643	4,213,736	3,593,719	620,018	180,668	2,249,241	87.95%
HG007	12.6	BCFTools	4,532,191	4,044,040	3,562,660	481,380	122,394	365,755	86.68%
		GATK4	4,692,527	4,060,472	3,548,979	511,493	101,163	530,891	86.94%
		DeepVariant	6,716,326	4,247,514	3,597,227	650,288	170,251	2,298,563	87.78%

^a^ Total called variants.

^b^ Identified as PASS, by Variant Transformer in case of BCFTools, and GATK4, or Identified as PASS, by DeepVariant filter.

## Discussion

The development and evaluation of our Transformers-based framework for variant refinement demonstrate the value of applying Transformer architectures to genomic post-processing tasks. This framework demonstrates an impressive variant refinement accuracy of 89.26%, which not only outperforms conventional refinement methods but also aligns closely with contemporary AI-based tools. By conceptualizing each variant record as a structured “sentence” and treating refinement as a classification problem, we illustrate how methods originally designed for natural language processing can be repurposed for genomics. This framework provides a scalable and adaptable foundation for improving variant quality, especially in challenging scenarios such as low-coverage sequencing.

VariantTransformer’s effectiveness in processing—a technique borrowed from NLP (Vaswani et al., 2017) — highlights its ability to handle the complex patterns inherent in genomic sequence data. By interpreting these patterns, the framework distinguishes true genetic variants from technical artifacts (Brown et al., 2020) with high efficiency, achieving ROC AUC scores that affirm its capacity to differentiate variant classes across all thresholds, thus enhancing its utility in varied analytical scenarios.

The aim of this work is to introduce a flexible and reproducible machine-learning framework for variant filtering, rather than a fixed benchmarking pipeline. Although GIAB data were used to derive high-quality labels, we did not treat the GIAB high-confidence BED as a constraint for formal FP/FN counting. Instead, we prioritized internal consistency by using the same positional labeling strategy during training and evaluation.

This design allows VariantTransformer to function as a general framework that can be adapted to any truth set or benchmarking paradigm. Users who require GIAB-compliant BED-restricted evaluation or genotype/allele/local matching through tools such as hap. py or rtg vcfeval can seamlessly integrate those components into the same methodology.

The framework’s success can be attributed to several key factors. First, the framework substantially improves the performance of existing variant calling pipelines. When applied to BCFTools and GATK4 outputs, the framework increased accuracy from ∼78 to 83% (heuristic filtering) to 86%–87%, approaching DeepVariant’s accuracy while reducing FPs. This balance between accuracy and precision highlights the value of post-calling refinement: whereas heuristic thresholds are brittle and overly sensitive to data sparsity, Transformers can dynamically learn the relationships among features, enabling more nuanced decisions. Second, modifications to the default BERT model parameters, specifically, reductions in hidden size and the number of attention heads, have tailored the model to handle the specific complexity of genomic data while optimizing computational efficiency (Vaswani et al., 2017). Finally, the MCC values highlight the framework’s balanced accuracy, considering both positive and negative classes, which is essential for applications in genomic studies where both sensitivity and specificity are critical (Chicco and Jurman, 2020). The ROC AUC scores further affirm the framework’s exceptional capability to distinguish between the variant classes across all thresholds, emphasizing its effectiveness in various scenarios (Fawcett, 2006).

While VariantTransformer was compared primarily against default filtering strategies from conventional callers (BCFTools and GATK4), an AI-based variant caller (DeepVariant), and an AI-based somatic refinement tool (DeepSVR), it is important to note that these tools differ in scope and intended use. Established refinement methods, such as GATK Variant Quality Score Recalibration (VQSR) or other ML-based filters, represent natural baselines for comparison; however, VQSR is not optimized for low-coverage data and can be unstable when applied outside its intended parameter range. Furthermore, DeepVariant incorporates its own AI-based filtering mechanism within its calling pipeline, making its results inherently post-processed and therefore not directly comparable to standalone refinement frameworks such as VariantTransformer. Accordingly, our choice of baselines reflects the focus on low-coverage, general-purpose refinement rather than direct benchmarking against high-coverage or internally filtered variant-calling pipelines. This clarification ensures that the reported improvements are interpreted within the context of the tool’s design and application domain.

When comparing VariantTransformer to DeepVariant, it is important to recognize the fundamental difference in their design and objectives. DeepVariant operates as a full variant caller that processes raw reads, while VariantTransformer functions purely as a post-calling refinement module. The two therefore differ substantially in computational scope: DeepVariant performs end-to-end inference, whereas VariantTransformer acts only on already-called variants. Our comparison is therefore conceptual rather than computational, highlighting the complementary roles of the two approaches. VariantTransformer enhances the output of conventional callers with minimal additional computational cost, improving accuracy and precision—particularly in low-coverage data—without replacing the upstream variant-calling process. Together, these approaches illustrate how AI-driven refinement can augment the performance of established tools, providing a practical and efficient complement to state-of-the-art AI-based variant callers.

While DeepVariant achieved slightly higher overall accuracy in some cases (87.95% for HG006 and 87.78% for HG007 with a difference of less than 1% when compared to VariantTransformer), it also presented a significantly higher rate of FPs for both samples. For sample HG006, DeepVariant had approximately 40% more FPs than BCFTools and approximately 29.38% more FPs than GATK4. For sample HG007, DeepVariant had approximately 35.07% more FPs than BCFTools and approximately 27.11% more FPs than GATK4. However, it is important to note that DeepVariant calls a larger number of total variants (∼6.6 million versus ∼4.5 million for GATK4 and BCFTools), which accounts for its higher absolute FP counts. When normalized by total calls, DeepVariant’s FP rate (21.62% for HG006 and 22.09% for HG007) is actually lower than those of GATK4 (44.94% and 49.07%) and BCFTools (53.65% and 56.89%). This normalization is not intended as a cross-caller accuracy comparison, but rather to contextualize absolute FP counts relative to each tool’s total number of emitted calls. These results are consistent with the precision values summarized in Table 3, providing a comprehensive view of each method’s performance. This comparison underscores VariantTransformer’s efficiency in minimizing incorrect variant identifications—a crucial advantage in genomic analytics—while acknowledging that some apparent FP from DeepVariant may arise from its higher sensitivity in challenging genomic regions that are incompletely represented in current benchmark truth sets (Wagner et al., 2022). However, it is also important to note that a reduction in FP does not universally indicate superior biological accuracy. In certain contexts, particularly when truth sets are incomplete, overly conservative models may classify genuine variants as FNs. Thus, VariantTransformer’s strength lies in offering a balanced refinement strategy that enhances call reliability without sacrificing sensitivity. Users who require representation-normalized comparisons may integrate GA4GH tools such as hap.py or vcfeval without altering the overall workflow.

Furthermore, the comparison with DeepSVR (Ainscough et al., 2018), a deep learning model developed to automate somatic variant refinement, underscores the distinct design objectives of the two frameworks rather than a direct performance comparison. DeepSVR classified SNPs into “Pass”, “Fail”, or “Ambiguous” with Fliess’ Kappa statistics (McHugh, 2012) indicating fair agreement (Ainscough et al., 2018). DeepSVR is designed and trained with a small training set (41,000 variants) of deep coverage (300–1,000X) cancer-type samples and is limited to SNP classification, requiring extensive data preprocessing before model input. In contrast, VariantTransformer was designed as a caller-agnostic post-calling refinement framework applicable to both SNPs and InDels, optimized for low-coverage germline data, and capable of operating directly on standard VCF files without additional preprocessing. These differences highlight that while DeepSVR is specialized for high-depth cancer genomics, VariantTransformer provides a more generalizable and lightweight approach suitable for a broader range of variant-calling scenarios, particularly those involving large-scale or population-level sequencing data (a detailed comparison of DeepSVR and VariantTransformer is presented in Supplementary Table S2).

While VariantTransformer achieves high accuracy, we must acknowledge that recent precisionFDA challenge participants have demonstrated superior F1 scores using higher coverage data (>30X), achieving over 99% in some cases. The framework’s strength lies in its balanced performance across various genomic contexts, particularly for low-coverage data, where precision and recall are paramount. This makes VariantTransformer a complementary tool rather than a replacement for existing high-coverage-focused AI-based callers.

Beyond performance, the most significant contribution of this work lies in its generality. The framework is not tied to a specific caller, sequencing platform, or coverage level. While demonstrated here on low-coverage Illumina datasets with GATK4 and BCFTools, the workflow is modular and can be adapted to other variant callers. Likewise, the Transformer backbone is flexible: while we instantiated it using a BERT-based model, other Transformer variants (e.g., RoBERTa, ALBERT, or lightweight domain-specific Transformers) could be employed with minimal modifications. This adaptability positions the framework as a general blueprint for integrating Transformer architectures into genomic refinement tasks.

However, there are limitations to our framework. One limitation of VariantTransformer is its current optimization for data generated from GATK and BCFTools. Although these tools remain standard in genomic studies, exploring the framework’s performance with VCFs generated by other modern callers like Clair3 or DeepVariant is crucial for generalizing its application. Future studies will focus on testing VariantTransformer’s adaptability to these tools and determining any necessary adjustments in preprocessing or feature engineering. Moreover, the training dataset, derived mainly from well-characterized genomic regions provided by the GIAB consortium, might not fully represent the diversity of genomic scenarios encountered in wider research and clinical contexts. Moreover, the framework configuration is primarily optimized for data generated from Illumina platforms. Future studies could expand the framework’s training scope to include more diverse and challenging genomic landscapes, potentially enhancing its applicability and accuracy in a wider array of genomic settings.

## Conclusion

In this study, we introduced a Transformers-based framework for genetic variant refinement and demonstrated its effectiveness in improving post-calling accuracy and precision across standard variant calling pipelines. By leveraging self-attention to capture dependencies among variant features, the framework consistently outperformed default heuristic filters in BCFTools and GATK4, raising accuracy from ∼78 to 83% to 86%–87% on independent GIAB samples. Although DeepVariant achieved slightly higher accuracy (∼88%), our framework reduced FPs, underscoring its strength in balancing accuracy with reliability. Importantly, VariantTransformer is not designed to replace variant callers but to serve as a complementary refinement layer that can be integrated downstream of any variant-calling pipeline to enhance call confidence and reproducibility. The pretrained model provided in this study is suitable for datasets that closely resemble the conditions under which it was developed, namely Illumina short-read low-coverage WGS processed with BCFTools or GATK4. For datasets that differ substantially in platform, coverage, or variant-calling pipeline, users may obtain improved performance by retraining or fine-tuning VariantTransformer on an appropriate truth set. This provides flexibility for deployment across diverse experimental settings. The framework is not limited to specific callers or datasets: it is modular, caller-agnostic, and adaptable to diverse sequencing contexts. This positions it as a generalizable blueprint for integrating Transformer architectures into genomic workflows, offering a flexible solution for low-coverage data and beyond. As sequencing continues to expand into population-scale and clinical settings, such frameworks will be essential for ensuring that downstream analyses are built on a foundation of robust and trustworthy genetic data.

## Data Availability

The complete dataset utilized in this study is accessible via the NIST GIAB FTP site (https://ftp-trace.ncbi.nlm.nih.gov/giab/ftp/data/). All the resources related to the analyses, including preprocessing code, training and testing code, and the trained framework, are available at https://github.com/Omar-Abd-Elwahab/VariantTransformer. This repository also offers guidance on using the framework for filtering VCFs, adapting it to other sequencing technologies, or integrating it into different variant calling pipelines. All VCF files used in training and evaluation, including the input callsets and the VariantTransformer output VCFs, have been deposited in Zenodo and are accessible under DOI: https://doi.org/10.5281/zenodo.17794617. These files are provided to support full reproducibility of the analyses.
