# Supplementary material for: A Transformers-based framework for refinement of genetic variants
Source: Front Bioinform. 2026 Jan 5;5:1694924. doi: 10.3389/fbinf.2025.1694924 (PMC12813134; doi:10.3389/fbinf.2025.1694924)
Supplement: Supplementary file 1 [file DataSheet1.pdf]

Supplementary Table S1. Training parameters employed during model development and final parameters used in the fine-tuned model. Default refers to pre-defined model parameters from Hugging Face documentation, while Model represents tuned parameters.

**Parameters**	**Default**	**VariantTransformer**
**Vocab_size**	30,522	30,567
**Never_split**	False	VCF unique words
**Hidden_size**	768	256
**num_hidden_layers**	12	8
**num_attention_heads**	12	8
**intermediate_size**	3072	512
**max_position_embeddings**	512	256
**classifier_dropout**	Optional (zero)	0.1

Supplementary Table S2. Comparison between DeepSVR and VariantTransformer models.

**Model**	**DeepSVR**	**VariantTransformer**
General functionality	Filters SNPs from specific cancer types samples	Filters SNPs and InDels from any raw VCF file with no preprocessing
Variant type	SNPs only	SNPs and InDels
Training size	41,000 variants	2,000,000 variants
Model's spectrum	Limited cancer types	Any sample
Training data preparation	Manual review of a small number of variants from cancer cases	Benchmark GIAB truth sets
Input data type	Requires preprocessing to a specific format with +50 features	No preprocessing: Any VCF file produced from a conventional variant calling pipeline (i.e. BCFTools and GATK4)
Testing data depth of coverage	Deep coverage of 312X up to 1,000X for some cases	Low coverage of 10X to 13.6X
